# Knowledge and Attitude Toward Esthetic Dentistry and Smile Perception

**DOI:** 10.7759/cureus.46043

**Published:** 2023-09-27

**Authors:** Alaa I Mannaa

**Affiliations:** 1 Restorative Dentistry, King Abdulaziz University, Jeddah, SAU

**Keywords:** restorative dentistry, esthetic dentistry, self-perception, smile perception, students vs professionals, dental treatments, smile attitude, smile knowledge

## Abstract

Introduction: A smile is important in human communication and is increasingly valued in modern society. The perception of smile attractiveness is subjective and depends on many factors.

Aim: This study aimed to evaluate and compare knowledge and attitude related to esthetic dentistry in dental students versus dental interns, general dentists, and dental specialists, examine the self-perception of their smile, and identify parameters that influence smile perception.

Methods: This cross-sectional study was conducted at King Abdulaziz University Dental Hospital in Jeddah, Saudi Arabia. Participants included dental students, interns, general practitioners, and specialists. They completed an electronic questionnaire covering demographics, knowledge about esthetic dentistry, attitudes toward smile perception, and previous dental treatments. Data analysis involved descriptive statistics, bivariate analysis, and chi-square tests.

Results: A total of 275 individuals participated in our study. The study found that dental students' exposure and awareness of esthetic dentistry topics increased with academic progression. Gender, age, and marital status influenced self-perception and smile evaluation. More females perceived gender as an influencing factor in smile evaluation, while more males believed in the existence of an ideal smile. A substantial portion of the sample had undergone dental treatments, with no significant income-related disparities observed.

Conclusion: This study highlights differences in knowledge and attitudes among dental students and professionals. Dental education appears to impact students' exposure to esthetic dentistry concepts. Moreover, gender, age, and marital status influence self-perception and evaluation of others' smiles.

## Introduction

The smile, an emblem of warmth and connection, holds profound significance in human communication. Consequently, modern society is placing increasing value on dental esthetics due to its ability to complement facial beauty [1]. The positive psychosocial effect gained by such pleasant facial esthetics accounts for the desire of patients to seek esthetic dental treatment [2]. This need for esthetic changes is often self-perceived and ultimately affects an individual’s self-confidence and quality of life [3].

The perception of beauty or attractiveness is very subjective and governs an individual’s perception of esthetics, depending on several factors including gender, age, ethnicity, socioeconomic status, marital status, level of education, occupation, familial influence, cultural exposure, and social media [4-6]. Within the field of dentistry, a natural smile is a complex expression influenced by dental structure, facial muscles, and emotions [7]. Its esthetics encompasses a multitude of factors that contribute to the overall perception of smile attractiveness and guide dental interventions [8]. Therefore, a comprehensive understanding of smile esthetics is essential for dental students in order to make them competent practitioners aware of the available esthetic treatment modalities and their respective indications [1]. Additionally, dental students should be aware of the differences in smile perception between dental professionals and laypeople to help them better understand their patients’ needs and expectations and plan optimal treatments.

In esthetic dentistry, smile design and analysis have relied on somewhat predefined and accepted scientific concepts that have been presented in a very static manner with specific measurements for form, color, and position of various smile elements. On the other hand, smile perception is a very dynamic concept that changes over time and is influenced by many factors. Social media for example has played a major role in the recent exponential growth of idealized smiles, dramatically influencing people’s self-perception of their own smiles as well as their rating of other individuals’ smiles [9]. Smile analysis and design have often been based on principles aiming toward achieving an esthetic, beautiful, ideal, or attractive smiles. However, esthetics is a broad zone composed of ranges of esthetic values for smile design. In light of this concept, it would be of interest to define the factors that influence people’s self-perception of their smiles and how they rate other individuals’ smiles.

Not much research has looked into dental students’ self-perception of their own smiles [5,10-13]. Furthermore, no studies have compared self-perception of smiles between students and dental professionals. To address this research gap, our study aims to evaluate and compare knowledge and attitudes related to esthetic dentistry in dental students versus dental interns, general dentists, and dental specialists, examine the self-perception of their smile, and identify the parameters that influence smile perception.

## Materials and methods

This cross-sectional study was conducted at King Abdulaziz University Dental Hospital (KAUDH) in Jeddah, Saudi Arabia (Research Ethics Committee at King Abdulaziz University Faculty of Dentistry (REC-KAUFD), approval number: #42548259, date: 22/12/2020).

Participants for this study were randomly selected from both staff and students present at the hospital clinics, ensuring a diverse representation. The inclusion criteria encompassed individuals who were currently enrolled as dental students, serving as interns, or practicing dentistry as graduates, whether in the capacity of general practitioners or specialists. No specific exclusion criteria were employed in this study.

The study was based on an electronic questionnaire consisting of four main sections.

Demographics 

This section involved questions about gender, age group, social status, income, academic year, and specialty (if applicable).

Knowledge of esthetic dentistry

This section was completed by students and interns and comprised two parts.

Part (a)

Part (a) included three closed-ended questions responded to with yes/no (Supplementary Table 2):

Q.1: About topics related to esthetic dentistry that were mentioned/covered in undergraduate education including anterior composite restorations, smile analysis, smile design, natural layering technique, direct and indirect veneers, teeth bleaching, micro-, macro-, and mega-abrasion, and enamel recontouring.

Q.2: About terms that were commonly used to describe a smile in undergraduate education including normal/abnormal, pleasant/unpleasant, beautiful/ugly, acceptable/unacceptable, attractive/non-attractive, ideal/imperfect, natural/artificial (un-natural), and perfect/defective (imperfect).

Q.3: About esthetic dentistry terms addressed during undergraduate dental studies including golden proportion, golden percentage, golden ratio, midline shift, diastema, gingival display, gingival symmetry/asymmetry, incisal symmetry, incisal embrasures, gingival margin design, gingival embrasures, gummy smile, incisal angulation, buccal corridor, smile arc, smile line, black triangles, crowding, spacing, incisal anatomy/design, canine characterization, teeth shade, and teeth morphology.

Part (b)

Part (b) included eight questions/statements focusing on factors considered in smile assessment, the level of knowledge in smile analysis and design, the existence of an ideal smile, the role of gender in smile perception, the difference between a beautiful and natural smile, and the multifactorial nature of smile esthetics and how it is defined by a predetermined set of factors. A five-point Likert response scale was used (Supplementary Table 3).

Attitude toward smile perception

This section was completed by all study participants and consisted of two parts.

Part (a)

This included two closed-ended questions responded to with yes/no (Supplementary Table 4).

Q.4: About factors influencing one’s own smile rating such as personal opinions and opinions of family, friends, peers/colleagues, public, and dental care providers as well as the smile of idols/celebrities/actors, televised media, audible media (radio), printed media (newspaper/magazine), advertisements, and social media platforms.

Q.5: About factors used in rating other people’s smile such as teeth color, shape, alignment, size, protrusion, cleanliness, and luster as well as gingival display, color, and lip thickness.

Part (b)

Part (b) included five statements focusing on rating one’s smile as natural and/or beautiful, keenness to dental care, self-confidence and embarrassment based on teeth appearance, and desire to have a natural, beautiful, and/or attractive smile. A five-point Likert response scale was used (Supplementary Table 5).

Previous esthetic dental treatments and cost expenditure

This section was completed by all study participants. This section focused on gathering information regarding previous esthetic dental treatment, motives behind it, and cost spent on it (Supplementary Table 6).

Participants were advised to complete the questionnaire using a tablet or laptop. Prior to commencement, all participants provided their digital consent, acknowledging the preservation of their anonymity and the freedom to withdraw from the survey at any time without any negative consequences or penalties.

The questionnaire was generated and administered through SurveyMonkey® (SurveyMonkey, Momentive, California, USA). IBM SPSS Statistics for Windows, Version 28 (Released 2021; IBM Corp., Armonk, New York, United States). was used for statistical analysis. The dependent variables were the participant’s responses in sections 2-4 as described earlier. The independent variables included study group, age, gender, social status, income, and specialty. To quantify the participants' responses to the survey, two sets of data were utilized as follows:

The first set of data was based on a scoring system specifically devised for the closed-ended knowledge and attitude questions (part (a) for each respectively, Q 1-5), where the mean score was computed for each participant. The scores were calculated by summing the topics/terms/factors to which a participant responded with “yes” and dividing it by the total number of answers for that specific question, thereby generating a score ranging from 0 to 1. A higher score indicated that a participant had encountered a greater number of topics, a wider range of terms, or utilized more factors for smile rating.

The second set of data included the responses utilizing the five-point Likert scale in parts (b) of the knowledge and attitude sections as well as the section on previous esthetic dental treatments and cost expenditure.

The study participants were divided into four categories: dental students, dental interns, general dentists, and dental specialists. For the knowledge section, the dental students were further subdivided into two groups according to their academic year into preclinical (3rd year) and clinical (4th, 5th, and 6th years), and the dental interns were added as a third subdivision (because undergraduate dental students in Saudi Arabia do not graduate until a final internship year is completed). Statistical analysis performed included descriptive statistics presented as frequencies/percentages and bivariate analysis (point-biserial correlation) to explore potential correlations between responses to questions in the knowledge and attitude sections and included the following paired analyses: a) Responses of dental students to knowledge part (a) questions (using the first data set) correlated with gender, social status, and academic year, b) responses to attitude part (a) questions (using the first data set) correlated with gender, social status, age group, income, and study group, c) interrelationship among the five scores for students and interns, and d) interrelationship between the two attitude scores for all participants, and the Chi-square test to examine the differences between the study categories, salary, and responses to the section related to previous esthetic dental treatments and cost (using the second data set).

## Results

In this cross-sectional study, a total of 275 individuals participated in our survey. Descriptive statistics are summarized in Table 1. Regarding part (a) of the knowledge of esthetic dentistry section, the majority of the correlations related to Q1 exhibited a direct relationship with the academic year (p < 0.05), meaning that the students were exposed to more esthetic dentistry topics and smile descriptive terms as they progressed to higher academic levels. On the other hand, Q2 yielded a solitary significant interaction, highlighting an elevated use of terms like "beautiful" and "ugly" to characterize smiles among students in advanced academic years (P < 0.05). As for Q3, an inverse correlation with social status was noticed (p < 0.05), meaning that a stronger correlation was observed with married students rather than their single counterparts and terms such as "gummy smiles", "buccal corridor", "smile line", "spacing", "teeth shading", and "teeth morphology". As for part (b), significant direct proportionate interactions were observed in relation to the academic year (p < 0.05).

**Table 1:** Descriptive statistics of the study participants

Variable	Frequency (n)	Percent (%)
Gender		
Male	151	54.9
Female	124	45.1
Age (years)		
<24	120	43.6
25-39	105	38.2
>40	50	18.2
Social status		
Single	142	51.6
Married	122	44.4
Divorced/Widowed	11	4
Income (Saudi Riyals)		
No income	49	17.8
<5000	59	21.5
5000 – 10,999	59	21.5
11,000 – 29,999	50	18.2
>30,000	58	21.1
Study group		
Student	65	23.6
Intern	74	26.9
General Dentist	52	18.9
Specialist	84	30.5
Specialty (for specialists)		
Restorative Dentistry	13	4.7
Endodontics	11	4
Prosthodontics	10	3.6
Orthodontics	14	5.1
Periodontics	18	6.5
Oral maxillofacial MF Surgery	7	2.5
Pedodontics	5	1.8
Other specialty	6	2.2
Academic year (for students)		
Preclinical (3rd year)	12	4.4
Clinical (4th, 5th & 6th years)	53	19.3
Internship	74	26.9

The attitude section of our survey was completed by all study participants. Regarding Q4, numerous interactions were associated with gender and age (P < 0.05), meaning that more factors influenced the females’ self-perception of smiles. Additionally, as people get older, they seem to become kinder in rating their own smiles. As for Q5, the interactions were primarily directly linked with age (P < 0.05), showing that as people age, they tend to use more factors to judge others' smiles. Gender also emerged as a prevailing factor in the interactions of the Likert-scale questions, exhibiting significant correlations favoring either males or females, as well as correlations with age, study group, and income (P < 0.05). More females and married people agreed with the statement "I find my smile natural." As for believing their smile to be beautiful, neither of the genders answered with yes significantly more than the other. However, more females believed their smile to affect their confidence, while more males felt embarrassed by their smiles.

We generated three scores based on the main knowledge questions (Q1-3) and two scores based on the main attitude questions (Q4 & 5). When we examined the relationship between the five question scores, we found some statistically significant patterns. First, there was a direct relationship between the first knowledge question (Q1) and the second and third knowledge questions, as well as the second attitude question (Q2, Q3, Q5) (P < 0.01). Furthermore, both the third knowledge question and the first attitude question (Q3, Q4) showed positive correlations with both the second knowledge question (Q2) and the second attitude question (Q5) (p < 0.01).

Regarding prior dental treatments, our results indicate that nearly half of our sample have undergone dental treatments. However, chi-square tests yielded no significant findings between the questions within this category and any of the demographic variables (p > 0.05).

## Discussion

The objective of this cross-sectional study aimed to delve into the perception of dental students regarding smile, encompassing their own, as well as comparisons with those of dental interns, general dental practitioners, and dental specialists. The null hypothesis posited that there would be no discernible disparity in smile perception between students and their professional counterparts. Our statistical findings, however, led to the rejection of the null hypothesis in several instances.

We posed three primary knowledge questions (Q1-3), each designed to gauge dental students' familiarity with distinct aspects of esthetic dentistry, encompassing esthetic dentistry topics, descriptive terminology for smiles, and esthetic dentistry terms. The outcomes of our findings revealed a predominant trend: a positive correlation in exposure to smile-related topics and associated terms as students progressed to higher academic levels. This trend aligned with our initial expectations. Notably, in both Q1 and Q3, which centered around smile-related topics and terms, advanced students such as interns naturally exhibited elevated exposure owing to the progressive nature of their curriculum and educational journey. On the other hand, Q2 yielded a solitary significant interaction, highlighting an elevated use of terms like "beautiful" and "ugly" to characterize smiles among students in advanced academic years. This observation warrants attention due to the correlation identified in Likert-scale questions pertaining to knowledge. Specifically, a noteworthy link emerged between higher-grade students and the belief in the multifactorial nature of smiles. Paradoxically, this finding contradicts the essence of a multifactorial assessment, which shouldn't be predicated on polarized extremes. This incongruence finds resonance in multiple psychological studies, which underscore the tendency to resort to such generalized terms when describing esthetics [14,15]. Conversely, a subset of interactions in Q3 displayed a stronger correlation with married students rather than their single counterparts. Notably, terms such as "gummy smiles," "buccal corridor," "smile line," "spacing," "teeth shading," and "teeth morphology" exhibited this pattern. Worth highlighting is that these correlations didn't relate to the student’s acceptance of the mentioned traits but rather to their coverage during the students' academic tenure. This pattern might stem from the potential influencing effect of certain personal attributes, like what a married person might see in his partner, which could attenuate the perception of traits that might otherwise be construed as deviating from the norm [16]. Examining this phenomenon in-depth, it's plausible that married individuals possessed an increased awareness of these traits even before encountering the corresponding terminology, potentially leading to their retention. This is especially pertinent considering the prevalence of some of these traits [17-19]. Another significant finding is that while more females perceived gender as a factor influencing smile evaluation, more males believed that there’s an ideal smile. This could be linked to the enhanced creative ability and vivid imagery often associated with women. Such cognitive attributes could potentially contribute to a broader spectrum of what is considered “ideal” [20].

As for the attitude, we asked two main questions (Q 4-5) to figure out what affects how people rate their own smiles and the smiles of others. In Q4, findings showed that as people get older, they seem to become kinder in rating their own smiles. However, in Q5, with others' smiles, results showed that as people age, they tend to use more factors to judge others' smiles. This is different from some previous studies that showed that older people were less critical, while others showed no age effect [21-24]. Our study's population, dentists and dental students, probably explains this difference, since they progress from students to dentists and specialists over time. In terms of the Likert-scale questions about attitudes, the statement "I find my smile natural" is connected to females and married people, while also being directly correlated with age, study group, and income. When we asked our participants if they "believe their smile to be beautiful," neither of the genders answered yes significantly more than the other. However, more females believed their smile to affect their confidence, while more males felt embarrassed by their smiles. This disparity aligns with a body of prior research, including some meta-analyses, showing that females tend to smile more, following social norms that expect them to be more expressive [25,26].

Our results indicate that nearly half of our sample have undergone dental treatments. Although income disparities exist within the context of Saudi Arabia, a nation marked by significant socioeconomic variations, we were unable to refute the null hypothesis that there is no effect of income level on dental treatments and how much is spent on it. These findings contradict previously published literature, which has associated lower income and wealth strata with compromised oral health and encountered financial barriers to dental care [27-30]. Our sample’s distinct composition, drawn exclusively from individuals with a professional background in dentistry, has likely contributed significantly to an elevated awareness of dental conditions and treatment modalities. To investigate this hypothesis further, prospective research should probe the income's impact on a more heterogeneous sample.

## Conclusions

In this cross-sectional study involving 275 participants, our findings revealed notable trends in dental students' perception of smiles and attitudes toward esthetic dentistry. As students advanced academically, their exposure to esthetic dentistry topics increased, but some trends, like the use of polarized terms, emerged paradoxically. Gender and age differences in attitude were evident, with older individuals becoming kinder in self-rating and more critical in assessing others' smiles. These insights contribute to our understanding of smile perception among dental professionals. However, the generalizability of the current study results is compromised due to the inclusion of a convenience sample drawn from one dental institute.

## Appendices

**Table 2:** Knowledge among study groups (three main questions)

Question	Preclinical students n = 12	Clinical students n = 53	Interns n = 74
(Q1) Which topics related to aesthetic dentistry were mentioned/covered in your educational curriculum?			
Anterior composite restorations			
Yes	5 (41.7%)	44 (83%)	53 (71.6%)
No	7 (58.3%)	9 (17%)	21 (28.4%)
Smile analysis & smile design			
Yes	4 (33.3%)	37 (69.8%)	53 (71.6%)
No	8 (66.7%)	16 (30.2%)	21 (28.4%)
Natural layering technique			
Yes	6 (50%)	22 (41.5%)	40 (54.1%)
No	6 (50%)	31 (58.5%)	34 (45.9%)
Direct & indirect veneers			
Yes	3 (25%)	34 (64.2%)	56 (75.7%)
No	9 (75%)	19 (35.8%)	18 (24.3%)
Teeth bleaching			
Yes	4 (33.3%)	35 (66%)	52 (70.3%)
No	8 (66.7%)	18 (34%)	22 (29.7%)
Micro-, macro- & mega-abrasion			
Yes	6 (50%)	30 (56.6%)	49 (66.2%)
No	6 (50%)	23 (43.4%)	25 (33.8%)
Enamel recontouring			
Yes	4 (33.3%)	19 (35.8%)	16 (21.6%)
No	8 (66.7%)	34 (64.2%)	58 (78.4%)
(Q2) Which terms were most commonly used to describe a smile?			
Normal/Abnormal			
Yes	5 (41.7%)	30 (56.6%)	37 (50%)
No	7 (58.3%)	23 (43.4%)	37 (50%)
Pleasant/Unpleasant			
Yes	5 (41.7%)	19 (35.8%)	25 (33.8%)
No	7 (58.3%)	34 (64.2%)	49 (66.2%)
Beautiful/Ugly			
Yes	0 (0%)	14 (26.4%)	28 (37.8%)
No	12 (100%)	39 (73.6%)	46 (62.2%)
Acceptable/Unacceptable			
Yes	5 (41.7%)	26 (49.1%)	33 (44.6%)
No	7 (58.3%)	27 (50.9%)	41 (55.4%)
Attractive/Non-attractive			
Yes	4 (33.3%)	17 (32.1%)	28 (37.8%)
No	8 (66.7%)	36 (67.9%)	46 (62.2%)
Ideal/Imperfect			
Yes	4 (33.3%)	23 (43.4%)	31 (41.9%)
No	8 (66.7%)	30 (56.6%)	43 (58.1%)
Natural/Artificial(un-natural)			
Yes	3 (25%)	22 (41.5%)	32 (43.2%)
No	9 (75%)	31 (58.5%)	42 (56.8%)
Perfect/Defective (Imperfect)			
Yes	1 (8.3%)	19 (35.8%)	21 (28.4%)
No	11 (91.7%)	34 (64.2%)	53 (71.6%)
(Q3) Were any of these aesthetic dentistry terms addressed during your studies?			
Golden proportion			
Yes	2 (16.7%)	33 (62.3%)	48 (64.9%)
No	10 (83.3%)	20 (37.7%)	26 (35.1%)
Golden percentage			
Yes	3 (25%)	31 (58.5%)	44 (59.5%)
No	9 (75%)	22 (41.5%)	30 (40.5%)
Golden Ratio			
Yes	7 (58.3%)	27 (50.9%)	51 (68.9%)
No	5 (41.7%)	26 (49.1%)	23 (31.1%)
Midline/Midline shift			
Yes	3 (25%)	42 (79.2%)	52 (70.3%)
No	9 (75%)	11 (20.8%)	22 (29.7%)
Diastema			
Yes	1 (8.3%)	37 (69.8%)	54 (73%)
No	11 (91.7%)	16 (30.2%)	20 (27%)
Gingival display			
Yes	2 (16.7%)	40 (75.5%)	56 (75.7%)
No	10 (83.3%)	13 (24.5%)	18 (24.3%)
Gingival symmetry/ Asymmetry			
Yes	5 (41.7%)	34 (64.2%)	53 (71.6%)
No	7 (58.3%)	19 (35.8%)	21 (28.4%)
Incisal Symmetry			
Yes	5 (41.7%)	29 (54.7%)	42 (56.8%)
No	7 (58.3%)	24 (45.3%)	32 (43.2%)
Incisal embrasures			
Yes	2 (16.7%)	30 (56.6%)	45 (60.8%)
No	10 (83.3%)	23 (43.4%)	29 (39.2%)
Gingival margin design			
Yes	2 (16.7%)	30 (56.6%)	42 (56.8%)
No	10 (83.3%)	23 (43.4%)	32 (43.2%)
Gingival embrasures			
Yes	3 (25%)	29 (54.7%)	45 (60.8%)
No	9 (75%)	24 (45.3%)	29 (39.2%)
Gummy smile			
Yes	3 (25%)	35 (66%)	48 (64.9%)
No	9 (75%)	18 (34%)	26 (35.1%)
Incisal angulation			
Yes	2 (16.7%)	24 (45.3%)	37 (50%)
No	10 (83.3%)	29 (54.7%)	37 (50%)
Buccal corridor			
Yes	3 (25%)	30 (56.6%)	42 (56.8%)
No	9 (75%)	23 (43.4%)	32 (43.2%)
Smile arc			
Yes	2 (16.7%)	28 (52.8%)	42 (56.8%)
No	10 (83.3%)	25 (47.2%)	32 (43.2%)
Smile line			
Yes	3 (25%)	31 (58.5%)	40 (54.1%)
No	9 (75%)	22 (41.5%)	34 (45.9%)
Black triangles			
Yes	1 (8.3%)	27 (50.9%)	42 (56.8%)
No	11 (91.7%)	26 (49.1%)	32 (43.2%)
Crowding			
Yes	5 (41.7%)	34 (64.2%)	47 (63.5%)
No	7 (58.3%)	19 (35.8%)	27 (36.5%)
Spacing			
Yes	3 (25%)	31 (58.5%)	44 (59.5%)
No	9 (75%)	22 (41.5%)	30 (40.5%)
Incisal anatomy/design			
Yes	3 (25%)	29 (54.7%)	39 (52.7%)
No	9 (75%)	24 (45.3%)	35 (47.3%)
Canine characterization			
Yes	5 (41.7%)	14 (26.4%)	30 (40.5%)
No	7 (58.3%)	39 (73.6%)	44 (59.5%)
Teeth shade			
Yes	3 (25%)	35 (66%)	45 (60.8%)
No	9 (75%)	18 (34%)	29 (39.2%)
Teeth morphology			
Yes	3 (25%)	31 (58.5%)	39 (52.7%)
No	9 (75%)	22 (41.5%)	35 (47.3%)

**Table 3:** Knowledge among study groups (Likert-scale questions)

Question	Preclinical students n = 12	Clinical students n = 53	Interns n = 74
I base my assessment of smiles on the aesthetic terms previously mentioned (refer to Q1). These lectures have influenced the factors I utilize for evaluating smiles.			
Strongly agree	1 (8.3%)	11 (20.8%)	10 (13.5%)
Agree	4 (33.3%)	22 (41.5%)	47 (63.5%)
Neutral	4 (33.3%)	15 (28.3%)	13 (17.6%)
Disagree	3 (25%)	4 (7.5%)	3 (4.1%)
Strongly disagree	0 (0%)	1 (1.9%)	1 (1.4%)
I regard my knowledge in smile analysis as			
Extensive	0 (0%)	7 (13.2%)	11 (14.9%)
Good	4 (33.3%)	19 (35.8%)	26 (35.1%)
Moderate	3 (25%)	20 (37.7%)	30 (40.5%)
Limited	2 (16.7%)	7 (13.2%)	6 (8.1%)
Very Limited	3 (25%)	0 (0%)	1 (1.4%)
I regard my knowledge in smile design as			
Extensive	1 (8.3%)	5 (9.4%)	5 (6.8%)
Good	6 (50%)	11 (20.8%)	20 (27%)
Moderate	3 (25%)	18 (34%)	31 (41.9%)
Limited	2 (16.7%)	17 (32.1%)	15 (20.3%)
Very Limited	0 (0%)	2 (3.8%)	3 (401%)
Evaluating smiles relies on predetermined factors			
Strongly agree	0 (0%)	6 (11.3%)	10 (13.5%)
Agree	6 (50%)	19 (35.8%)	37 (50%)
Neutral	3 (25%)	20 (37.7%)	18 (24.3%)
Disagree	3 (25%)	6 (11.3%)	7 (9.5%)
Strongly disagree	0 (0%)	2 (3.8%)	2 (2.7%)
There's an ideal smile			
Strongly agree	0 (0%)	6 (11.3%)	6 (8.1%)
Agree	5 (41.7%)	22 (41.5%)	36 (48.6%)
Neutral	4 (33.3%)	13 (24.5%)	21 (28.4%)
Disagree	3 (25%)	11 (20.8%)	7 (9.5%)
Strongly disagree	0 (0%)	1 (1.9%)	4 (5.4%)
Smile aesthetics/attractiveness is multifactorial			
Strongly agree	1 (8.3%)	8 (15.1%)	20 (27%)
Agree	6 (50%)	25 (47.2%)	43 (58.1%)
Neutral	3 (25%)	14 (26.4%)	9 (12.2%)
Disagree	1 (8.3%)	5 (9.4%)	2 (2.7%)
Strongly disagree	1 (8.3%)	1 (1.9%)	0 (0%)
There's a difference between a beautiful smile and a natural smile			
Strongly agree	1 (8.3%)	9 (17%)	14 (18.9%)
Agree	5 (41.7%)	24 (45.3%)	38 (51.4%)
Neutral	3 (25%)	14 (26.4%)	17 (23%)
Disagree	2 (16.7%)	4 (7.5%)	5 (6.8%)
Strongly disagree	1 (8.3%)	2 (3.8%)	0 (0%)
Gender plays a role in smile evaluation			
Strongly agree	0 (0%)	11 (20.8%)	17 (23%)
Agree	7 (58.3%)	20 (37.7%)	40 (54.1%)
Neutral	3 (25%)	15 (28.3%)	14 (18.9%)
Disagree	1 (8.3%)	5 (9.4%)	2 (2.7%)
Strongly disagree	1 (8.3%)	2 (3.8%)	1 (1.4%)

**Table 4:** Attitude among the study groups (two main questions)

Question	Dental student n = 63	Dental intern n = 74	General practitioners n = 41	Specialist n = 82
(Q4) What factors influence the rating of your smile?				
Personal opinion				
Yes	42 (64.6%)	41 (55.4%)	29 (55.8%)	51 (60.7%)
No	21 (35.4%)	33 (44.6%)	12 (44.2%)	31 (39.3%)
Family opinion				
Yes	20 (30.8%)	18 (24.3%)	12 (23.1%)	16 (19%)
No	43 (69.2%)	56 (75.7%)	29 (76.9%)	66 (81%)
Friends’ opinion				
Yes	19 (29.2%)	25 (33.8%)	12 (23.1%)	17 (20.2%)
No	44 (70.8%)	49 (66.2%)	29 (76.9%)	65 (79.8%)
Peers/colleagues opinion				
Yes	19 (29.2%)	20 (27%)	7 (13.5%)	12 (14.3%)
No	44 (70.8%)	54 (73%)	34 (86.5%)	70 (85.7%)
Public opinion				
Yes	14 (21.5%)	15 (20.3%)	6 (11.5%)	11 (13.1%)
No	49 (78.5%)	59 (79.7%)	35 (88.5%)	71 (86.9%)
Your dentist's opinion				
Yes	21 (32.3%)	34 (45.9%)	15 (28.8%)	27 (32.1%)
No	42 (67.7%)	40 (54.1%)	26 (71.2%)	55 (67.9%)
The smile of my idols/celebrities/actors				
Yes	15 (23.1%)	13 (17.6%)	3 (5.8%)	7 (8.3%)
No	48 (76.9%)	61 (82.4%)	38 (94.2%)	75 (91.7%)
Televised media				
Yes	9 (13.8%)	5 (6.8%)	2 (3.8%)	7 (8.3%)
No	54 (86.2%)	69 (93.2%)	39 (96.2%)	75 (91.7%)
Audible media (radio)				
Yes	10 (15.4%)	9 (12.2%)	0 (0%)	9 (10.7%)
No	53 (84.6%)	65 (87.8%)	41 (100%)	73 (89.3%)
Printed media (newspaper/magazine)				
Yes	13 (20%)	5 (6.8%)	2 (3.8%)	5 (6%)
No	50 (80%)	69 (93.2%)	39 (96.2%)	77 (94%)
Advertisements				
Yes	12 (18.5%)	4 (5.4%)	1 (1.9%)	5 (6%)
No	51 (81.5%)	70 (94.6%)	40 (98.1%)	77 (94%)
Social media platforms				
Yes	12 (18.5%)	4 (5.4%)	1 (1.9%)	5 (6%)
No	51 (81.5%)	70 (94.6%)	40 (98.1%)	77 (94%)
(Q5) What factors do you use in rating someone else’s smile?				
Teeth color				
Yes	36 (55.4%)	42 (56.8%)	27 (51.9%)	50 (59.5%)
No	27 (44.6%)	32 (43.2%)	14 (48.1%)	32 (40.5%)
Teeth shape				
Yes	31 (47.7%)	37 (50%)	24 (46.2%)	46 (54.8%)
No	32 (52.3%)	37 (50%)	17 (53.8%)	36 (45.2%)
Lips thickness				
Yes	18 (27.7%)	21 (28.4%)	17 (23.7%)	28 (33.3%)
No	45 (72.3%)	53 (71.6%)	24 (76.3%)	54 (66.7%)
Teeth alignment				
Yes	42 (64.6%)	49 (66.2%)	28 (53.8%)	47 (56%)
No	21 (35.4%)	25 (33.8%)	13 (46.2%)	35 (44%)
Teeth size				
Yes	28 (43.1%)	39 (52.7%)	25 (48.1%)	38 (45.2%)
No	35 (56.9%)	35 (47.3%)	16 (51.9%)	44 (54.8%)
Gingival display				
Yes	26 (40%)	50 (67.6%)	27 (51.9%)	43 (51.2%)
No	37 (60%)	24 (32.4%)	14 (48.1%)	39 (48.8%)
Gingival color				
Yes	30 (46.2%)	40 (54.1%)	20 (38.5%)	37 (44%)
No	33 (53.8%)	34 (45.9%)	21 (61.5%)	45 (56%)
Teeth protrusion				
Yes	26 (40%)	45 (60.8%)	23 (44.2%)	32 (38.1%)
No	37 (60%)	29 (39.2%)	18 (55.8%)	50 (61.9%)
Teeth cleanliness				
Yes	34 (52.3%)	45 (60.8%)	25 (48.1%)	49 (58.3%)
No	29 (47.7%)	29 (39.2%)	16 (51.9%)	33 (41.7%)
Teeth luster				
Yes	17 (26.2%)	27 (36.5%)	12 (23.1%)	31 (36.9%)
No	46 (73.8%)	47 (63.5%)	29 (76.9%)	51 (63.1%)

**Table 5:** Attitude among the study groups (Likert-scale questions)

Question	Dental student n = 63	Dental intern n = 74	General practitioners n = 41	Specialist n = 82
I find my smile natural				
Strongly agree	8 (12.7%)	16 (21.6%)	14 (34.1%)	24 (29.3%)
Agree	32 (50.8%)	48 (64.9%)	24 (58.5%)	48 (58.5%)
Neutral	11 (17.5%)	7 (9.5%)	3 (7.3%)	7 (8.5%)
Disagree	8 (12.7%)	3 (4.1%)	0 (0%)	2 (2.4%)
Strongly disagree	4 (6.3%)	0 (0%)	0 (0%)	1 (1.2%)
I find my smile beautiful				
Strongly agree	12 (19%)	14 (18.9%)	7 (17.1%)	17 (20.7%)
Agree	21 (33.3%)	47 (63.5%)	29 (70.7%)	50 (61%)
Neutral	18 (28.6%)	8 (10.8%)	3 (7.3%)	13 (15.9%)
Disagree	7 (11.1%)	3 (4.1%)	2 (4.9%)	2 (2.4%)
Strongly disagree	5 (7.9%)	2 (2.7%)	0 (0%)	0 (0%)
I tend to overemphasize my dental care routine				
Strongly agree	5 (8.2%)	10 (13.9%)	2 (5.4%)	8 (10.4%)
Agree	18 (29.5%)	49 (68.1%)	18 (48.6%)	35 (45.5%)
Neutral	19 (31.1%)	8 (11.1%)	7 (18.9%)	21 (27.3%)
Disagree	14 (23%)	3 (4.2%)	9 (24.3%)	12 (15.6%)
Strongly disagree	5 (8.2%)	2 (2.8%)	1 (2.7%)	1 (1.3%)
The appearance of my teeth affects my self-confidence				
Strongly agree	10 (16.4%)	16 (22.2%)	8 (21.6%)	17 (22.1%)
Agree	23 (37.7%)	35 (48.6%)	19 (51.4%)	27 (35.1%)
Neutral	19 (31.1%)	15 (20.8%)	5 (13.5%)	14 (18.2%)
Disagree	6 (9.8%)	4 (5.6%)	3 (8.1%)	14 (18.2%)
Strongly disagree	3 (4.9%)	2 (2.8%)	2 (5.4%)	5 (6.5%)
I am embarrassed by the appearance of my teeth				
Strongly agree	5 (8.2%)	9 (12.5%)	4 (10.8%)	10 (13%)
Agree	12 (19.7%)	19 (26.4%)	9 (24.3%)	21 (27.3%)
Neutral	20 (32.8%)	18 (25%)	6 (16.2%)	10 (13%)
Disagree	14 (23%)	13 (18.1%)	10 (27%)	16 (20.8%)
Strongly disagree	10 (16.4%)	13 (18.1%)	8 (21.6%)	20 (26%)

**Table 6:** Previous dental treatment among the study groups

Question	Dental students	Dental interns	General practitioners	Specialists
I want my smile to be:				
Natural	14 (22.2%)	22 (29.7%)	10 (24.4%)	19 (23.2%)
Beautiful	10 (15.9%)	14 (18.9%)	3 (7.3%)	13 (15.9%)
Attractive	20 (31.7%)	9 (12.2%)	8 (19.5%)	10 (12.2%)
All of the above	15 (23.8%)	27 (36.5%)	18 (43.9%)	37 (45.1%)
Indifferent	4 (6.3%)	2 (2.7%)	2 (4.9%)	3 (3.7%)
Did you receive any treatment for your teeth or your smile?				
Yes	27 (44.3%)	26 (36.1%)	20 (54.1%)	39 (50.6%)
No	34 (55.7%)	46 (63.9%)	17 (45.9%)	38 (49.4%)
If yes, what was the treatment?				
Bleaching	10 (34.5%)	6 (19.4%)	4 (18.2%)	9 (18%)
Restorations	3 (10.3%)	2 (6.5%)	1 (4.5%)	6 (12%)
Veneers	5 (17.2%)	2 (6.5%)	3 (13.6%)	1 (2%)
Crowns/bridges	1 (3.4%)	1 (3.2%)	1 (4.5%)	2 (4%)
Implants	1 (3.4%)	1 (3.2%)	0 (0%)	3 (6%)
Gingival surgery	1 (3.4%)	1 (3.2%)	1 (4.5%)	5 (10%)
Braces	7 (24.1%)	16 (51.6%)	10 (45.5%)	22 (44%)
Botox/filler	1 (3.4%)	2 (6.5%)	2 (9.1%)	2 (4%)
What motived you to seek such treatment?				
Dental issues/conditions	9 (31%)	11 (36.7%)	5 (23.8%)	11 (21.5%)
Marriage	3 (10.3%)	0 (0%)	3 (14.3%)	9 (17.6%)
To keep up with fashion	6 (20.7%)	1 (3.3%)	0 (0%)	5 (9.8%)
My family/friends encouraged me	3 (10%)	2 (6.7%)	1 (4.8%)	7 (13.7%)
To improve my smile appearance (no dental issues present)	8 (27.6%)	16 (53.3%)	12 (57.1%)	19 (37.3%)
How much in average did you spend (Saudi Riyals)?				
<1000	11 (35.5%)	6 (20%)	6 (30%)	12 (23.5%)
1000-5000	9 (29%)	9 (30%)	5 (25%)	14 (27.5%)
5000-10,000	7 (22.6%)	7 (23.3%)	4 (20%)	15 (29.4%)
>10,000	4 (12.9%)	8 (26.7%)	5 (25%)	10 (19.6%)

## References

[1] Manipal S, Mohan CS, Kumar DL, Cholan PK, Ahmed A, Adusumilli P (2014). The importance of dental aesthetics among dental students assessment of knowledge. J Int Soc Prev Community Dent.

[2] Klages U, Claus N, Wehrbein H, Zentner A (2006). Development of a questionnaire for assessment of the psychosocial impact of dental aesthetics in young adults. Eur J Orthod.

[3] Phillips C, Beal KN (2009). Self-concept and the perception of facial appearance in children and adolescents seeking orthodontic treatment. Angle Orthod.

[4] Geron S, Atalia W (2005). Influence of sex on the perception of oral and smile esthetics with different gingival display and incisal plane inclination. Angle Orthod.

[5] da Silva G, de Castilhos ED, Masotti AS, Rodrigues-Junior SA (2012). Dental esthetic self-perception of Brazilian dental students. Revista Sul-Brasileira de Odontologia.

[6] Vallittu PK, Vallittu AS, Lassila VP (1996). Dental aesthetics--a survey of attitudes in different groups of patients. J Dent.

[7] Manjula WS, Sukumar MR, Kishorekumar S, Gnanashanmugam K, Mahalakshmi K (2015). Smile: a review. J Pharm Bioallied Sci.

[8] Simonsen R (2015). Chapter 2 - Ethics considerations in esthetic dentistry. Principles and Practice of Esthetic Dentistry.

[9] Abbasi MS, Lal A, Das G (2022). Impact of social media on aesthetic dentistry: general practitioners' perspectives. Healthcare (Basel).

[10] Al-Saleh S, Abu-Raisi S, Almajed N, Bukhary F (2018). Esthetic self-perception of smiles among a group of dental students. Int J Esthet Dent.

[11] El Mourad AM, Al Shamrani A, Al Mohaimeed M, Al Sougi S, Al Ghanem S, Al Manie W (2021). Self-perception of dental esthetics among dental students at King Saud University and their desired treatment. Int J Dent.

[12] Sipiyaruk K, Chanvitan P, Kongton N, Runggeratigul P, Niyomsujarit N (2022). The impact of smile appearance and self-perceived smile attractiveness on psychological well-being amongst dental undergraduates. Int J Clin Dent.

[13] Subait AA, Ali A, Hammad ZA, Alrumaih A, Al-Malki A, Al-faqih A, El-Metwally A (2016). Dental aesthetics and attitudes among University students in Saudi Arabia-a cross-sectional study. J Dent Oral Disord.

[14] Augustin MD, Wagemans J, Carbon CC (2012). All is beautiful? Generality vs. specificity of word usage in visual aesthetics. Acta Psychol (Amst).

[15] Jacobsen T, Buchta K, Kohler M, Schröger E (2004). The primacy of beauty in judging the aesthetics of objects. Psychol Rep.

[16] Bull R, Brooking J (1985). Does marriage influence whether a facially disfigured person is considered physically unattractive?. J Psychol.

[17] Almotairy N, Almutairi F (2022). A Nation-wide prevalence of malocclusion traits in Saudi Arabia: a systematic review. J Int Soc Prev Community Dent.

[18] de Brito ML, Silva ML, Carvalho BW, da Silva EM, de Lira A (2023). Prevalence and factors associated with gummy smile in adolescents: a cross-sectional analysis. Braz J Oral Sci.

[19] Elhiny O (2014). Prevalence of gummy smile in a sample of Egyptian population and laymen perception of its attractiveness. Egypt Orthod J.

[20] Forisha BL (1978). Creativity and imagery in men and women. Percept Mot Skills.

[21] Abu Alhaija ES, Al-Shamsi NO, Al-Khateeb S (2011). Perceptions of Jordanian laypersons and dental professionals to altered smile aesthetics. Eur J Orthod.

[22] Alsurayyi MA, Almutairi W, Binsaeed AI, Aldhuwayhi S, Shaikh SA, Mustafa MZ (2022). A cross-sectional online survey on knowledge, awareness, and perceptions of Hollywood Smile Among the Saudi Arabia population. Open Dent J.

[23] Ansari SH, Abdullah Alzahrani AA, Said Abomelha AM, Attia Elhalwagy AE, Mustafa Alalawi TN, Mahmoud Sadiq TW (2020). Influence of social media towards the selection of Hollywood smile among the University students in Riyadh city. J Family Med Prim Care.

[24] Mokhtar HA, Abuljadayel LW, Al-Ali RM, Yousef M (2015). The perception of smile attractiveness among Saudi population. Clin Cosmet Investig Dent.

[25] Hall JA (1990). Nonverbal Sex Differences: Accuracy of Communication and Expressive Style. https://psycnet.apa.org/record/1985-98125-000.

[26] LaFrance M, Hecht MA, Paluck EL (2003). The contingent smile: a meta-analysis of sex differences in smiling. Psychol Bull.

[27] Locker D, Maggirias J, Quiñonez C (2011). Income, dental insurance coverage, and financial barriers to dental care among Canadian adults. J Public Health Dent.

[28] Manski RJ, Moeller JF, Chen H, St Clair PA, Schimmel J, Pepper JV (2012). Wealth effect and dental care utilization in the United States. J Public Health Dent.

[29] Singh A, Peres MA, Watt RG (2019). The relationship between income and oral health: a critical review. J Dent Res.

[30] Watson MR, Manski RJ, Macek MD (2001). The impact of income on children's and adolescents' preventive dental visits. J Am Dent Assoc.

